# *In vitro* and *ex vivo* comparison of reactive oxygen-releasing granules for internal tooth bleaching

**DOI:** 10.3389/fdmed.2024.1447459

**Published:** 2024-08-29

**Authors:** Romy M. Mayer, Andrea Gubler, Thomas Attin, Matthias Zehnder

**Affiliations:** Clinic for Conservative and Preventive Dentistry, University of Zurich Center for Dental Medicine, Zurich, Switzerland

**Keywords:** bleaching, dental, hydrogen peroxide, carbamide peroxide, sodium percarbonate, sodium perborate

## Abstract

**Introduction:**

Traditionally, internal tooth bleaching was performed using sodium perborate slurries. These are banned in some areas for potential carcinogenic effects. More recently, highly concentrated hydrogen peroxide gels have been used, which may cause dentin degradation. Consequently, the search for ideal internal tooth bleaching agents is still on. This study compared pure ROS-releasing granules regarding their liberation of oxidizing species, pH induction, bleaching of blood-stained dentin, and effects on mechanical dentin properties.

**Materials and methods:**

The ROS-releasing granules under investigation were sodium perborate, carbamide peroxide, and sodium percarbonate in aqueous suspension (4:3, wt/wt). The bleaching efficacy of these suspensions was compared in blood-stained human dentin (*n* = 6) *ex vivo*. In addition, effects on mechanical dentin integrity were tested using bovine dentin beams (*n* = 9) exposed to a 3-point bending test (ISO 4049) after immersion in test suspensions or control solutions (35% H_2_O_2_ and physiological saline) for 1 week.

**Results:**

Granules release between 21.5% and 35.2% (wt/wt) of H_2_O_2_ equivalent. The sodium-containing granules (perborate and percarbonate) caused an alkaline pH of 10.3 and 10.6, respectively. The carbamide peroxide suspension was acidic (pH 3.9), as was the 35% H_2_O_2_ solution used as a control (pH 2.2). All the suspensions bleached the blood-stained dentin, albeit with a lesser overall effect by sodium percarbonate (one-way ANOVA and Tukey's HSD, *p* < 0.05). The acidic preparations caused a severe (over 50%) reduction in flexural strength of the dentin (*p* < 0.05 compared to physiological saline solution), while the alkaline counterparts did not.

**Conclusions:**

Sodium perborate granules in aqueous suspension combined good de-staining properties with limited untoward effects on dentin integrity. Further studies are required to identify alternative compounds with a lesser general health concern.

## 1 Introduction

Non-vital teeth containing either a necrotic pulp or a previously administered root filling can be discolored. The discoloration can stem from extravascular blood, root canal medicaments, or root filling materials (1). As the affected teeth require root canal treatment or are already root canal-treated, the intervention of first choice to reduce the discoloration is the so-called walking bleach method, in which a bleaching agent is placed into the pulp chamber of the discolored tooth crown (2, 3). This method, also referred to as internal or intracoronal tooth bleaching (4), appears to be safer than the in-office administration of highly concentrated bleaching agents in conjunction with heat or light, which have been suspected to cause external cervical root resorption (5, 6).

Traditionally, internal tooth bleaching protocols used aqueous suspensions of reactive oxygen species (ROS)-releasing granules, which were placed into the pulp chamber of a pulpless tooth, which was then sealed with a temporary material. The walking bleach technique was introduced by Spasser (7). He used a thick aqueous suspension of sodium perborate. Later, granular sodium perborate was also mixed with a hydrogen peroxide (H_2_O_2_) solution, and different granule/solution mixtures were tried out involving H_2_O_2_, carbamide peroxide, and sodium perborate, either as aqueous suspensions or in a polyethylene glycol (PEG) matrix (3, 8). As of more recently, highly concentrated H_2_O_2_ for internal bleaching is also available in gel form, i.e., in a PEG paste (9, 10). However, there are several aspects in the context of the walking bleach technique, which have not been discussed in sufficient detail or in the context of current legislature (11). A first concern is the Boron contained in sodium perborate. Sodium perborate has been banned from cosmetic products and dental bleaching agents in Europe for its potentially carcinogenic effect (12). On the other hand, gel-type medical devices for internal tooth bleaching containing plain H_2_O_2_ may also contain non-disclosed ingredients that may exert cytotoxic effects (13). H_2_O_2_ is acidic (14), and in bleaching agents has been associated with dentin degradation (15), potentially leading to a higher fracture propensity (16), and also external cervical root resorption (5).

Not only from a patient safety and regulatory, but also from a purely clinical perspective, it appears timely to search for alternatives to the current gold standards for internal tooth bleaching. It is easier and more practical to apply granules in aqueous suspension into an access cavity than a H_2_O_2_ solution or a PEG-based H_2_O_2_ paste, because the temporary filling is easier to apply (17). Thus, and considering that we are living in the age of ever-increasing regulation of medical devices (11), straight-forward preparations involving chemically pure granules and water may become *en vogue* again. In this context, granular carbamide peroxide or sodium percarbonate could be used as alternatives to sodium perborate for the walking bleach method (18). However, comparative data on these agents apart from enamel bleaching applications for external application (19) are sparse. As discussed above, there is a key chemical difference between the different preparations for internal tooth bleaching: aqueous H_2_O_2_ and carbamide peroxide are acidic, while sodium perborate is alkaline (20), and so is sodium percarbonate (because of its sodium release). Interestingly, alkaline bleaching agents appear to induce less structural changes in dentin than acidic preparations (9). Sodium perborate suspensions in water appear to be less aggressive on dentin than H_2_O_2_-containing formulations (4, 15). This may be why sodium perborate suspensions appear to still be popular for this purpose wherever they are not explicitly prohibited (8). However, alkaline ROS-releasing preparations have also shown to reduce the fracture strength of bovine teeth when administered as intra-coronal bleaching agents (21), and do not necessarily bleach any better than acidic counterparts at similar concentration (14, 22).

The goal of this *in vitro*/*ex vivo* study was to comparatively assess the chemistry and effects of acidic and alkaline ROS-releasing granules in the context of the walking bleach technique. Granular sodium perborate, carbamide peroxide, and sodium percarbonate were compared regarding their content of oxidative species and pH induction in aqueous suspension. Their bleaching kinetics were assessed in blood-stained human coronal dentin (23). Furthermore, potential untoward effects of these suspensions on the mechanical integrity of dentin were tested in a standardized three-point bending test (ISO 4049) using bovine dentin beams. Results were compared to those obtained with a 35% H_2_O_2_ solution. The Null hypothesis tested was that there were no differences between the bleaching effects and influence on mechanical dentin properties of the ROS-releasing granular suspensions under investigation and a 35% H_2_O_2_ control solution.

## 2 Materials and methods

### 2.1 Biological materials used in this study

This study used biological materials in the form of extracted third molars, excess human blood from donors, and bovine dentin from animals raised, kept, and slaughtered for food consumption. The use of these materials in this investigation was in line with local ethics guidelines (24, 25). The current work was neither considered to be a human nor an animal study by the local ethics committee (Kantonale Ethikkommission Zürich). Human specimens were anonymized, and informed consent was obtained from all donors that their extracted wisdom teeth were to be used for research in an anonymized manner. The human blood was excess material intended to create blood agar for microbiology. It contained saline, adenine, glucose and mannitol (26).

Human and bovine teeth used in this study were thoroughly cleaned from periodontal soft tissue remnants using scalers and scalpels under running tap water. Personnel handling these specimens applied all the necessary hygienic precautions. Subsequently, teeth were stored in a 0.5% Chloramine-T (Carl Roth, Karlsruhe, Germany) solution at 5°C in a refrigerator until further use.

### 2.2 Chemical assessments

All the ROS-containing granules under investigation were acquired from Sigma Aldrich (St. Louis, MO, USA): sodium perborate (NaBO_3_ tetrahydrate, purum p.a, Lot No. BCCJ8645), carbamide peroxide (CH_6_N_2_O_3_, urea hydrogen peroxide 97%, Lot No. BCCK3729), and sodium percarbonate (Na_2_CO_3_ trihydrate, Lot No. BCCK2793). For better readability, the names of these chemicals/granules rather than their chemical formulas are used in this text.

The weight content of H_2_O_2_ equivalent in the granules under investigation was determined by iodine titration (27). A standard protocol was followed (28). To that end, 200 mg of each agent was dissolved in 100 ml of Milli-Q water. The resulting solution was rendered acidic by adding sulfuric acid (H_2_SO_4_), and spiked with 1 g of potassium iodide (PanReac AppliChem 131542.1209, Darmstadt, Germany). Subsequently, 1 ml of a 3% ammonium molybdate catalyst solution (Sigma Aldrich) was added, and the solution was kept in the dark for 5 min for all the iodine to be liberated. This liberated iodine was then titrated immediately using a 0.1 M sodium thiosulfate (S_2_O_3_^2−^) solution (PanReac AppliChem 186987.1211) in a titration apparatus (665 Dosimat, Metrohm). One mole of H_2_O_2_ equaled 2 mole of S_2_O_3_^2−^ in this calculation (28). As soon as the color started to change, 2 ml a 0.2% starch solution was added for better detection of the endpoint (complete color loss of the solution).

Suspensions of 1 g of the granules under investigation were mixed with 0.75 ml of deionized water (Milli-Q, Merck, Rahway, NJ, USA) for all subsequent experiments. Granules to liquid mixtures were prepared using a precision balance (PM300, Mettler-Toledo, Greifensee, Switzerland). A solution with an iodometrically confirmed content of 35% H_2_O_2_ (Sigma Aldrich) was used as the positive control in all the experiments, a non-buffered physiological saline solution (0.9% NaCl, B. Braun, Melsungen, Germany) as the negative control. The pH values of these suspensions were measured using a calibrated pH electrode (6.0228.010, Metrohm, Herisau, Switzerland) attached to a pH measuring device (727 pH lab, Metrohm).

### 2.3 Power analysis for the use of human teeth

This was based on a former study comparing the bleaching effect between NaOCl and H_2_O_2_ solutions (14), and a comparable study on some of the suspensions under investigation (18). Based on the differences between groups and the standard deviations, an effect size of 1 was assumed, a total sample size of 25 (ANOVA, fixed effects, omnibus, one-way) was calculated (G*Power 3.1, Heinrich-Heine Universität, Düsseldorf, Germany). To get more robust data, the total sample size was increased to 30 (*n* = 6).

### 2.4 Bleaching of blood-stained human dentin

For these experiments, a published set-up was used (23). In brief, 30 intact human wisdom teeth extracted for reasons not related to this study were selected. Teeth were attached with their roots to a scanning electron microscopy stub using a denture resin (Paladur, Kulzer, Hanau, Germany) for saw cutting. Dentin discs of 4 mm thickness and 6.6 mm diameter were cut out using first a machine saw (Isomet Low Speed Saw, Speed 10, Buehler, Lake Buff, IL, USA) equipped with a diamond-coated disc (Diamond Cut-off Wheel, MOD13, Struers, Ballerup, Denmark) and then a trephine bur in a drill system (BFW 40/E, Proxxon, Föhren, Germany). These discs were then placed in cylindrical silicon molds of 6 mm height and 8.8 mm inner diameter and embedded in resin (Paladur, Kulzer) under vacuum with their coronal surface facing down. After polymerisation, the resin cylinders were trimmed down from the resin side to a standard height of 5 mm. The exposed (outer) dentin surface corresponding to the site of color assessment was polished using silicon carbide grinding paper (Prüfag, Schlieren, Switzerland) of 2,500 grit for 15 s followed by 4,000 grit for 40 s. Subsequently, a cylindrical reservoir was drilled into the resin cylinder from the other side using a carbide drill of 5 mm diameter (Holex, Munich, Germany) attached to the drilling machine (Proxxon BFW 40/E). Drilling depth was controlled visually with a marking on the drill and verified using a digital caliper (150 mm, Holex). This resulted in similar specimens with a standardized reservoir for the placement of human blood then the bleaching agents, and a dentin thickness of 1 mm (23). The outer rim of the resin reservoir was marked using a permanent pen for specimen placement in the spectrophotometer (see below). To open the dentinal tubules from the inside, the reservoir of the specimens was filled with 50 µl of a 17% EDTA solution, pH 8 (Kantonsapotheke, Zürich, Switzerland) for 5 s using a pipetter (Pipetman, 10–100 µl, Gilson, Madison, Wisconsin). The specimen was then rinsed with deionized water, and the process of EDTA demineralization was repeated twice. This was done immediately before the first color assessment and subsequent placement of the human blood (see below).

Color assessments were performed in in the CIEL*a*b* color space where the L* value indicates the white to black, a* the green to red, and b* the blue to yellow hue. The polished dentin surfaces were assessed using a black/white-calibrated spectrophotometer (Konica Minolta CM-2600d, Tokyo, Japan) connected to an external computer running the analysis software (Spectra Magic NX, version 2.8, Konica Minolta). Specimens were positioned on the device using a customized aluminum holder using their mark on the reservoir so that they could be reassessed in the same position. Measurements were performed in reflectance mode. Images were taken with a field of view of 3 mm (Target Mask A147, Konica Minolta) under simulated natural light illumination (D65).

The treatment side (reservoir) of the specimens was then filled with 50 µl of human blood. Specimens were then individually placed in 15 ml-centrifugation tubes (Greiner Bio-One, Frickenhausen, Germany) with their exposed dentin side facing down. Subsequently, specimens were centrifuged at 3,000 * g for 2 min (Heraeus Megafuge 8R Centrifuge, Thermo Scientific, Waltham, MA, USA), and then incubated (INCU-Line, VWR, Dietikon, Switzerland) at 37°C and 100% relative humidity for 3 weeks to increase discoloration (14, 23).

**Figure 1:**
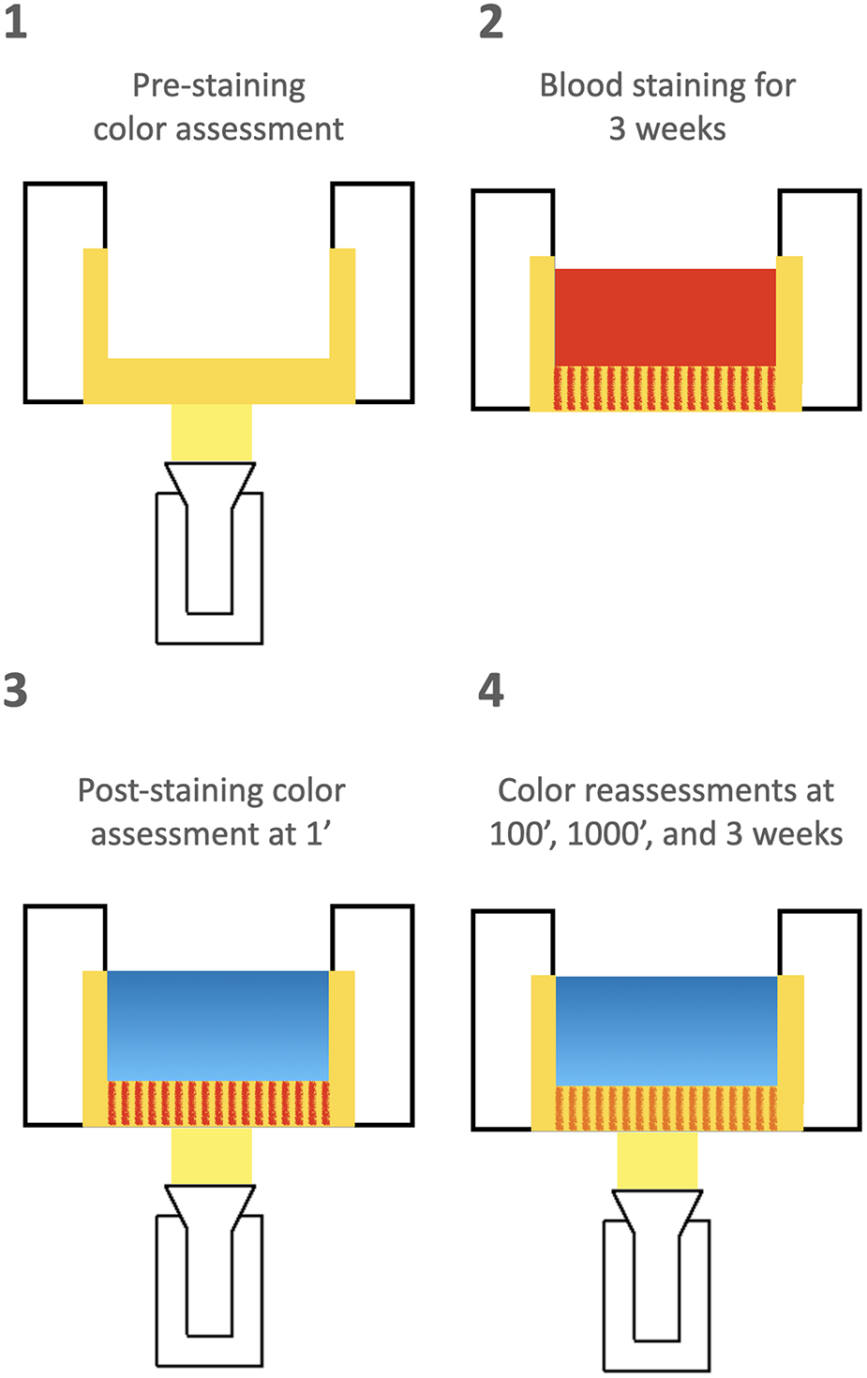
Schematic depiction of the timeline relating to the 5 color assessments that were performed in this study **(**panels **1**, **3**, and **4)**. Specimens consisted of human dentin from the roof of the pulp chamber embedded in resin. Red filling in reservoir: blood; blue filling: test suspensions or control solutions. Note that the test and control agents were kept in the reservoir for the whole study duration. Specimens were kept at 37°C in 100% relative humidity before and between measurements.

Then the 4:3 suspensions of the test powders under investigation in Milli-Q water were placed in the reservoir side of the specimens (*n* = 6) using a plastic spatula. The reservoir side was filled half-way. In the control specimens (*n* = 6), 50 µl of a the 35% H_2_O_2_ solution (positive control) or physiological saline solution was pipetted. Color was re-assessed immediately after the reservoirs had been filled with test or control agents as described above. Subsequently, the specimens were again incubated as described, and the dentin color was re-assessed after 100 min, 1,000 min, and 3 weeks (Figure 1).

### 2.5 Mechanical assessments

The effects of the test and control agents under investigation on mechanical dentin properties were assessed using standardized bovine dentin beams. The test was performed according to ISO 4049 (Chapter 7.11: Flexural Strength). However, the dentin beams differed in their dimensions from those recommended in the norm (which requests test specimens in the dimension of 25 mm * 2 mm * 2 mm). They were 16 mm * 0.8 mm * 1.2 mm. These dentin beams were cut from bovine incisor roots using a precision saw (Isomet Low Speed Saw, Buehler, Lake Buff, IL, USA). To that end, teeth were decoronated and attached to scanning electron microscopy stubs using methacrylate resin (Paladur, Kulzer, Hanau, Germany). The first section was in a longitudinal plane, resulting in three to four root sections of 1.2 mm thickness. Subsequently, these sections were embedded again as described, and beams of 0.8 mm width were dissected. Finally, the beams were adjusted to 16 mm in length using a disc saw in a dental laboratory hand piece. A total of 45 dentin beams were produced, and then randomly immersed in the three test suspensions or two control solutions (physiological saline as a positive and 35% H_2_O_2_ as a negative control, *n* = 9). Beams were immersed individually in an incubator at 37°C for 1 week.

The 3-point bending tests was performed in a universal testing machine (Zwick Roell, Ulm Germany) at a cross-head speed of 1 mm/min using two stilts at a distance of 10 mm and a point radius of 1 mm. Before testing, the exact dimension of the bovine dentin beams were measured using a digital caliper (Holex) and entered in the proprietary software program of the apparatus (testXpert lll, Zwick Roell). The outcome measurements were modulus of elasticity (GPa) and flexural strength (MPa).

### 2.6 Data presentation and analysis

Data pertaining to chemical measurements are presented as means and standard deviations to indicate the measurement error of the triplicate assessments.

Where not mentioned explicitly, L, a, and b data from the CIEL*a*b* color space are presented as delta values, indicating that each of these measurements was compared within specimens over time, thus reducing variability. Mean changes and standard deviations are presented. Data related to changes in the CIEL*a*b* color space as well as those relating to mechanical dentin properties were distributed evenly (Shapiro-Wilk test), and differences between treatment were compared by one-way ANOVA and Tukey's HSD, *p* < 0.05.

## 3 Results

### 3.1 Chemical assessments

The ROS-releasing granules under investigation had a similar content of oxidative species with a H_2_O_2_ equivalent between 21.5% and 35.2% (Table 1). However, they differed in their water solubility, with sodium perborate showing the lowest solubility by far. The pH in the aqueous suspensions was alkaline in the sodium containing formulations, and acidic in the carbamide peroxide suspension (Table 1). In the 35% H_2_O_2_ solution, used as a control in the following experiments, the pH was between 2.15 and 2.18 (triplicate measurement).

**Table 1:** Chemical parameters of the reactive oxygen-releasing powders under investigation.

Parameter	Sodium perborate	Carbamide peroxide	Sodium percarbonate
H_2_O_2_ equivalent content (wt/wt)	21.5 ± 0.0	35.2 ± 0.1	27.6 ± 0.1
Solubility (g/L) at 20°C	0.1 (0.01%)^a^	50 (5%)^a^	140 (14%)^a^
pH in suspension immediately after 30 min	10.25–10.29 10.20–10.21	3.92–3.96 3.91–3.96	10.60–10.60 10.63–10.67

Results represent means and standard deviations (H_2_O_2_ content) and ranges (pH values) of triplicate measurements.

^a^ This information was taken from the data sheet (Sigma Aldrich).

### 3.2 Blood staining and onset of de-staining effect

The blood staining caused a significant (*p* < 0.05) decrease in L* values from 67.8 ± 4.9 to 57.0 ± 5.0, with no difference between the specimens assigned to the different treatment groups.

The kinetics of the bleaching effect was then assessed by comparing the L* values between the first minute after applying the test and control substances into the simulated access cavities and the subsequent measuring points (Figure 1). Each specimen served as its own control, i.e., changes within each specimen were compared over time (Table 2). The 35% H_2_O_2_ solution caused a rapid bleaching effect, with significantly (*p* < 0.05) increased Delta L* values compared to the treatment with physiological saline solution already observed after 100 min. The suspensions of the sodium perborate and the carbamide peroxide powders reached a bleaching effect that was statistically similar to that of the 35% H_2_O_2_ solution after 1,000 min, while the corresponding treatment with the sodium percarbonate powder in suspension did not reach these levels, yet still bleached the blood-stained specimens (Table 2).

**Table 2:** Change of L* values from black to white in the CIELAB color space according to treatment and time of assessment.

Treatment	100 min	1,000 min
Non-buffered physiological saline (negative control)	0.1 ± 2.5^a^	–2.7 ± 3.4^a^
35% H_2_O_2_ (positive control)	11.6 ± 1.9^b^	18.3 ± 5.5^c^
Sodium perborate	0.6 ± 2.2^a^	10.0 ± 3.5^b,c^
Carbamide peroxide	3.3 ± 2.7^a^	10.9 ± 7.5^b,c^
Sodium percarbonate	4.0 ± 2.5^a^	8.3 ± 2.4^b^

Values correspond to difference in lightness between a treatment time and the initial value after placing the test or control agent; means and standard deviations (*n* = 6).

Shared superscript letters indicate that there were no difference between treatments at a given time of assessment (columns); one-way ANOVA and Tukey's HSD, *p* < 0.05.

### 3.3 Final color change from baseline

**Figure 2:**
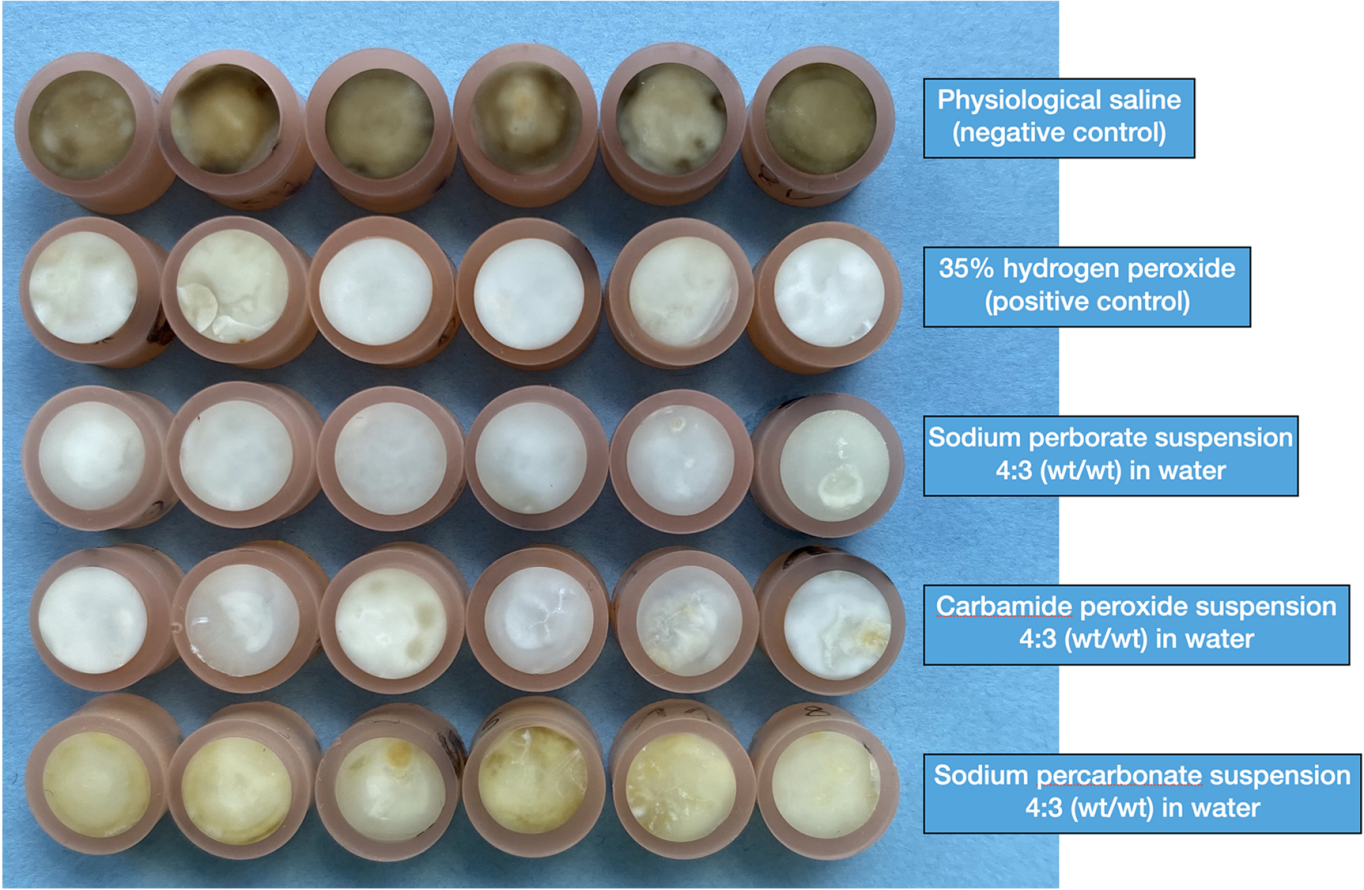
Photographic overview of all the blood-stained human dentin specimens under investigation. This photograph was taken after the 3-week staining and subsequent 3-week de-staining (treatment) period.

The specimens were re-assessed after 3 weeks of immersion with the test and control agents in their respective reservoirs. Compared to the L* values before blood staining, the 35% H_2_O_2_ solution had the strongest bleaching effect under current conditions (Table 3). All the bleaching agents rendered the specimens less red (Delta a*) and yellow (Delta b*). However, visually the sodium perborate and carbamide peroxide suspensions had a similar effect as the 35% H_2_O_2_ solution, while the sodium percarbonate suspension was less effective (Figure 2).

**Table 3:** Overall change in color of the specimens between the initial appearance (before blood staining) and after three weeks of staining and another three weeks of treatment.

Treatment	Delta L*	Delta a*	Delta b*
Non-buffered physiological saline (negative control)	−11.8 ± 5.9^a^	3.0 ± 1.0^a^	5.4 ± 5.9^a^
35% H_2_O_2_ (positive control)	10.0 ± 6.1^c^	−0.6 ± 0.7^b^	−8.0 ± 4.2^b^
Sodium perborate	2.0 ± 2.7^b^	−1.4 ± 1.3^b^	−11.4 ± 1.7^b^
Carbamide peroxide	3.3 ± 1.6^b,c^	−1.5 ± 1.3^b^	−11.6 ± 4.3^b^
Sodium percarbonate	−3.5 ± 2.4^b^	−1.1 ± 1.1^b^	−5.1 ± 3.1^b^

Shared superscript letters indicate that there were no difference between treatments at a given time of assessment (columns); one-way ANOVA and Tukey's HSD, *p* < 0.05.

Delta L* indicates black to white, Delta a* green to red, and Delta b* blue to yellow values in the CIELAB color space. Negative values indicate changes towards black, green, and blue values, respectively. Positive values indicate the corresponding changes towards white, red, and yellow. Means and standard deviations (*n* = 6).

### 3.4 Effects on mechanical dentin properties

The alkaline suspensions under investigation, i.e., sodium perborate and sodium percarbonate, increased the modulus of elasticity in the bovine dentin specimens slightly, while the hydrogen peroxide solution (pH 2.15–2.18) had the opposite effect (Table 4). These differences were even more pronounced on the flexural strength values, with the 35% H_2_O_2_ and the carbamide peroxide suspension causing a reduction of more than 50% compared to the control treatment with a physiological saline solution, while the alkaline suspensions had little to no negative effect (Table 4).

**Table 4:** Mechanical parameters of the bovine dentin beams after immersion in test and control solutions/suspensions for 1 week, means and standard deviations (*n* = 9).

Treatment	Modulus of elasticity (GPa)	Flexural strength (MPa)
Non-buffered physiological saline (positive control)	9.9 ± 2.2^a,b,c^	251.2 ± 36.1^a^
35% H_2_O_2_ (negative control)	7.4 ± 0.7^c^	82.7 ± 16.1^c^
Sodium perborate	11.3 ± 2.1^a,b^	211.9 ± 33.7^a,b^
Carbamide peroxide	8.5 ± 1.9^b,c^	116.9 ± 26.9^c^
Sodium percarbonate	11.4 ± 2.9^a^	203.1 ± 42.8^b^

Shared superscript letters indicate that there were no difference between treatments at a given time of assessment (columns); one-way ANOVA and Tukey's HSD, *p* < 0.05.

## 4 Discussion

In the current study, ROS-releasing granules in aqueous suspension were compared regarding their chemical parameters, bleaching effect on blood-stained human dentin, and induction of mechanical alterations in bovine dentin. Results strongly suggest that while their bleaching effects were similar, the alkaline suspensions containing sodium perborate or sodium percarbonate caused less mechanical integrity-reducing alterations in dentin compared to the acidic carbamide peroxide, or the 35% H_2_O_2_ solution that was used as a control.

This study is unique in that it took a wholistic look at the desired property of the chemicals under investigation, while considering one of their main untoward effects, namely dentin degradation. However, this investigation is limited by a multitude of factors. Even though natural substrates were used, this was still an *in vitro*/benchtop study, and no clinical conclusions should be drawn from these observations. The number of specimens (*n* = 6 for the bleaching experiment and *n* = 9 for the mechanical assessments) was limited for ethical reasons. However, such a low number of observations may hamper the possibility to detect subtle differences between treatment groups. Furthermore, the application of the test suspensions and the control solutions occurred in one single dose. In clinics, agents for internal tooth bleaching are changed at a 1-week interval (2, 8), a procedure that is not really rooted in clinical evidence (4). This was not done here, as we wanted to study the kinetics of the bleaching effect of one single application with different ROS-releasing powders in aqueous suspension. A further limitation of this study is that the ROS-releasing capacity of the granules under investigation was not assessed at the different time points. In contrast to earlier investigations, we did not mix the peroxide-releasing powders under investigation with a H_2_O_2_ solution, as we wanted to study their stand-alone efficacy and also potential detrimental effects on dentin. It should also be conceded that the medication-substrate ratio in the experiment on mechanical dentin properties was much higher than in clinics. Bovine dentin beams were immersed in the suspensions/solutions. Nevertheless, the results reported here are rather similar to those observed with human dentin beams (29, 30). Moreover, chemicals reducing the flexural strength of dentin in the current set-up also cause in increased fracture propensity of whole tooth roots under simulated clinical conditions (31).

The staining and subsequent bleaching effects of the agents under investigation measured in our model with a Delta L* of 10–15 units were very similar to those measured in extracted human premolars stained with human blood (18). The only difference between the data presented here and counterparts obtained in whole teeth is that the effects were observed earlier in our model. This can be explained by a lack of enamel covering the stained dentin in the model we used. Nevertheless, our data is confirmed by clinical studies, which showed that 35% H_2_O_2_ in pure water or a PEG matrix has an earlier onset, and also tends to bleach slightly better than suspensions of ROS-releasing granules under investigation (3, 32). This can be explained by the higher immediate availability and also the higher concentration of H_2_O_2_ in these applications (Table 1).

The chemical compounds under investigation, albeit all bleaching agents, differ chemically. Carbamide peroxide and sodium percarbonate are simple adducts with hydrogen peroxide, while sodium perborate consists of a cyclic core with two hydroxy groups attached to each boron. In view of the low solubility of the perborate granules in water, it is most intriguing how well (and quickly) they bleach. The explanation for this remains elusive, but is probably found in the complex dissociation and release of oxidative species that occurs when perborate is dissolved in water. Moreover, perborate is suspected to have an additional, non-oxidative bleaching effect. This, however, was not the topic of the current study. Because the European Union has declared borates as carcinogenic, mutagenic, or toxic for reproduction (CMR) in 2010, they have automatically been banned in dental products/medical devices (12), and a thorough investigation of their effects in the current context thus appears to be unnecessary. However, our results do confirm early observations from the laundry bleaching industry that sodium perborate causes little damage to organic moieties. This sets this compound apart from chlorine bleaches, which are much more aggressive. Historically, a multitude of substances has been used for internal tooth bleaching, with chlorine and peroxide/perborate-based agents as early contenders (33). Chlorine in the form of hypochlorite has a higher bleaching power than hydrogen peroxide (14). However, hypochlorite also has a much higher deproteinizing effect than hydrogen peroxide (34), and therefore a greater potential to make teeth prone to fracture (31) via its detrimental effects on the organic dentin matrix including the collagen (35). This is why aqueous calcium hypochlorite suspensions, which could technically be used for the internal bleaching process via their steady release of hypochlorite, have not been used for this process. Apparently, both low pH and hydrogen peroxide oxidation play a role in altering the ultrastructure of dentin during internal dental bleaching (9). The use of alkaline products may decrease such morphological alterations (4). This was confirmed in the current study, with the acidic preparations reducing the fractural strength of the dentin beams by more than half, and the alkaline suspensions having no such detrimental effect.

Overall, and taking together the results from the current efficacy and safety tests (and considering the fact that perborate is banned), sodium percarbonate stood out as an interesting alternative to more aggressive agents. This warrants further investigations with this compound, which was originally introduced to the endodontic literature in the year 2000 (18). However, clinical studies or commercially available products for internal tooth containing sodium percarbonate are missing, even though this compound is contained in tooth whitening strips and paint-on-whiteners (19, 36). Randomized clinical trials on the topic thus far have involved a commercial polyethylene glycol (PEG) gel containing 35% hydrogen peroxide (32), sodium perborate and carbamide peroxide suspensions in water or aqueous hydrogen peroxide (8).

## 5 Conclusions

Sodium percarbonate in granular form appears to be an alternative to sodium perborate to create slurries for internal tooth bleaching, as it does not contain any doubtful components and does considerably less damage to mechanical dentin integrity than acidic ROS-releasing preparations. However, its bleaching effect is inferior to that of sodium perborate, and therefore the search for the ideal internal tooth bleaching agent or compound continues.

## Data Availability

The raw data supporting the conclusions of this article will be made available by the authors, without undue reservation.
